# Impact of glucagon-like peptide 1 receptor agonist liraglutide and dipeptidyl peptidase-4 inhibitor sitagliptin on bowel cleaning and gastrointestinal symptoms in type 2 diabetes

**DOI:** 10.3389/fphar.2023.1176206

**Published:** 2023-04-06

**Authors:** Yan Tong, Jian Qing Huang, Yang Chen, Mei Tu, Wei Wang

**Affiliations:** Longyan First Affiliated Hospital of Fujian Medical University, Longyan, Fujian, China

**Keywords:** liraglutide, sitagliptin, bowel preparation quality, gastrointestinal discomfort, gastrointestinal motility

## Abstract

**Objective:** Glucagon-like peptide 1 receptor agonists (GLP-1 RAs) and dipeptidyl peptidase-4 inhibitors (DPP-4i) profoundly affect the gastrointestinal motor system, which may increase the incidence of inadequate bowel cleaning and gastrointestinal symptoms. Hence, this observational study mainly aimed to assess the influence of GLP-1 RAs liraglutide and DPP-4i sitagliptin on bowel preparation in type 2 diabetes (T2DM).

**Method:** This observational study consecutively enrolled T2DM scheduled for a colonoscopy. Participants were prospectively separated into the liraglutide group (*n* = 120), sitagliptin group (*n* = 120), and control group (*n* = 120) based on the current hypoglycemic regimen. 3L split-dose polyethylene glycol regimens were used for bowel preparation. Experienced gastrointestinal endoscopists conducted colonoscopies. Lawrance Bowel-Preparation Tolerability Questionnaire and Boston Bowel Preparation Scale (BBPS) were conducted to assess bowel cleaning quality, tolerability, and safety.

**Results:** The incidence of inadequate bowel cleaning was 17.5% in the liraglutide group, 20.5% in the sitagliptin group, and 21.7% in the control group. The difference among the three groups was not statistically significant (*p* = 0.927). Meanwhile, there were no significant differences in the mean BBPS, cecal intubation time, and polyp-detecting rates among the three groups (all *p* > 0.0.05). Nausea, vomiting, and bloating scores were increased in the liraglutide group compared with the other two groups (*p* < 0.05), whereas most were mild or very mild. Subgroup analyses showed that the incidence of inadequate bowel cleaning in T2DM with diabetic peripheral neuropathy (DPN) was increased in the liraglutide group compared with the sitagliptin group (61.3% vs. 32.1%, *p* = 0.022) and control group (61.3% vs. 32.8%, *p* = 0.025).

**Conclusion:** GLP-1RA liraglutide or DPP-4i sitagliptin did not significantly increase the incidence of inadequate bowel cleaning and gastrointestinal symptoms during bowel preparation. Liraglutide may increase the incidence of inadequate bowel preparation in patients with DPN. This study reveal that more attention and aggressive bowel preparation regimens should be given to the T2DM with DPN.

**Clinical Trial Registration:** (https://www.chictr.org.cn/index.aspx), identifier (ChiCTR2200056148).

Clinical Trial Registration: [website], identifier [registration number].

## Introduction

Diabetes mellitus (DM) is a metabolic disease mainly characterized by chronic hyperglycemia and a worldwide public problem threatening human health. According to the International Diabetes FederationDiabetes Atlas (10th edition) (Ogurtsova et al., 2022), the prevalence of DM is 10.5% in adults (20–79 years old) at present, and the majority may increase to 11.43% in 2030% and 12.2% in 2045. DM is closely related to the occurrence and development of digestive tumors that have been reported to be an independent risk factor for malignant colorectal tumors (Aleksandrova et al., 2011). In addition, DM is prone to gastrointestinal dysfunction. Nearly half suffer from gastrointestinal symptoms, including vomiting, nausea, constipation, diarrhea, fecal incontinence, or abdominal pain (Selbuz and Buluş, 2020; Sang et al., 2022). Colonoscopy has been widely used as a valuable tool for screening and treating colorectal diseases in the last 2 decades. Due to the high prevalence of digestive disease in a large number of diabetic populations, colonoscopy has been widely performed in routine clinical diagnosis and treatment of diabetes with gastrointestinal symptoms. Emerging evidence highlighted that some hypoglycemic agents could inhibit intestinal motility, which might affect the quality of bowel preparation and increase gastrointestinal symptoms (Chung et al., 2009; Gandhi et al., 2018).

Hypoglycemic agents such as sulfonylureas, thiazolidinediones, and insulin have rarely been reported to affect gastrointestinal motility. Few studies found that metformin and α-glucosidase inhibitors might affect gastrointestinal motility, but these effects are weak and easily affected by other intestinal hormones (Jalleh et al., 2022). Glucagon-like peptide 1 (GLP-1) is a kind of hormone secreted predominantly by the proximal small intestine that can reduce blood glucose by stimulating insulin release, decreasing gastric emptying, inhibiting food intake, glucagon secretion, and modulating rodent β-cell proliferation (Drucker, 2018). GLP-1 receptor agonists (GLP-1 RAs) are analogs of human native GLP-1 hormone that can increase plasma GLP-1 half-life and has widely used as hypoglycemic agents in T2DM (Wang et al., 2023). Besides the well-known hypoglycemic effect of GLP-1 RAs, increasing evidence showed that GLP-1 RAs also profoundly affect the gastrointestinal motor system (Marathe et al., 2011). In contrast, the impact of GLP-1 RAs on gut motility may vary with the change in intestinal segments (Nauck et al., 1997; Thazhath et al., 2016; Wegeberg et al., 2020). Endogenous GLP-1 is rapidly degraded by the dipeptidyl peptidase-4 (DPP-4), resulting in a short half-life. DPP-4 inhibitors (DPP-4i) can also reduce blood glucose by increasing endogenous GLP-1 concentrations that can also influence gastrointestinal motility (Deacon, 2020). Good diagnostic performance and fewer complication rates depend on good bowel cleaning quality. More studies have focused on the effects of GLP-1 RAs and DPP-4i on gastrointestinal motility at pharmacological concentrations and the motor actions of incretin-based therapies. In contrast, few studies have put insights into the potential effects of these agents on bowel cleaning quality and gastrointestinal discomfort during bowel preparation. Current guidelines did not address the recommendations of GLP-1 receptor agonists and DPP-4 inhibitors on bowel preparation. Hence, we established an observational study to assess the effects of GLP-1 RAs liraglutide and DPP-4i sitagliptin on bowel cleaning quality, tolerability, and safety.

## Materials and methods

### Patients and study design

**FIGURE 1:**
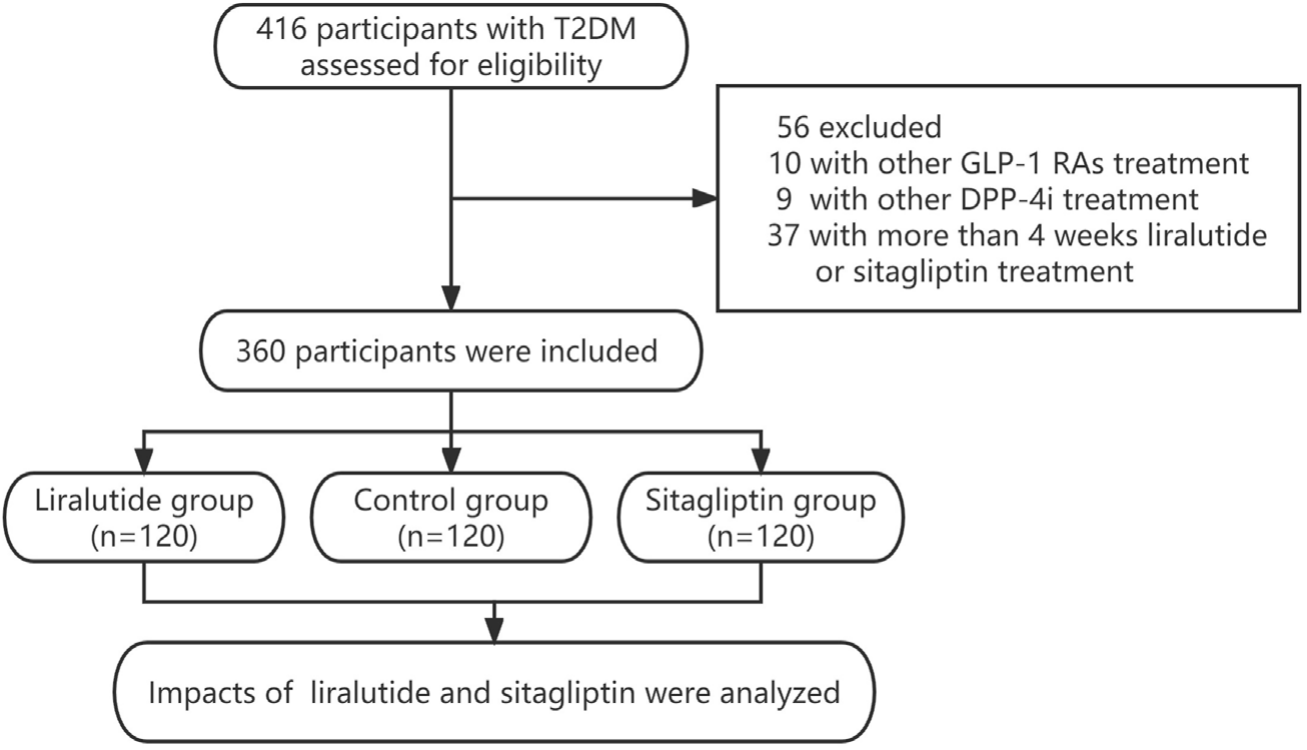
Flowchart describing the selection process of the study population in this study.

This prospective observational study enrolled T2DM from the Department of Endocrinology at the Longyan First Affiliated Hospital of Fujian Medical University, who screened for polyp requiring colonoscopy between January 2020 and December 2022. The study inclusion criteria were as follows: 1) poor glycemic control (HbA1c>7.0%) requires the addition of hypoglycemic drugs such as liraglutide or sitagliptin. 2) currently, with the treatment of liraglutide or sitagliptin (within 4 weeks). T2DM were excluded if they were as follows:1) Accompanied by severe chronic renal failure (CKD 3–5), heart failure (New York Heart Association Class II–IV), unstable angina, acute or chronic pancreatitis, serum electrolyte disturbances, and pregnancy. 2)History of gastrointestinal or other abdominal surgery. 3)Suspected gastrointestinal perforation, obstruction, and bleeding. 4) Long-term use of the laxative or prokinetic drug. 5) Currently using liraglutide or sitagliptin for more than 4 weeks. 6) Currently using other kinds of GLP-1 RAs or DPP-4i. 7) With fasting blood glucose≥ 11.1 mmol/L or acute diabetic complications like hyperglycemia, hyperosmolar syndrome, or diabetic ketoacidosis during bowel preparation. All procedures were conducted in compliance with the Declaration of Helsinki. All procedures were performed in compliance with the Declaration of Helsinki. This study was approved by the Ethical Committee of Longyan First Affiliated Hospital of Fujian Medical University (LY-2020–072). All patients gave written informed consent. We assumed that the incidence of adequate bowel cleaning in control group might range from 80% to 85%, and the liraglutide group may have a 5%–10% reduction. Based on the statistical results of SASS software, the study population consisted of the liraglutide group (n = 120), sitagliptin group (n = 120), and control group (n = 120). The flowchart describing the selection process of the study population is summarized in Figure 1.

### Anthropometric and laboratory assessments

The clinical data were collected by a trained interviewer through a standard questionnaire that consisted of age, duration of diabetes, history of diseases that could interfere with bowel cleaning quality, current or prior use of drugs, smoking, drinking, and gastrointestinal symptoms. Information was also obtained through a review of medical records and laboratory data. Height and weight were measured to the nearest 0.1 cm and 0.1 kg. Patients wear hospital gowns and bare feet. BMI was calculated as the weight divided by the square of height (kg/m2). The following laboratory assessments were measured by standard methods using fasting venous blood samples, which were taken between 8 and 9 a.m. after fasting overnight. Creatinine, alanine aminotransferase, and electrolyte were measured by an auto-biochemical analyzer (Roche Diagnostics Corporation). Glycosylated hemoglobin (HbA1c) was evaluated by high-performance liquid chromatography with a D10 set (Bio-RAD). In addition, retinal photography, electromyography, and urinary microalbumin to creatinine ratio (ACR) were also performed to screen diabetes-related complications.

### Bowel preparation

All participants received the same bowel preparation regimens and were instructed to eat a semi-fluid low-fiber diet 2 days before the colonoscopy. During bowel preparation and colonoscopy, all participants were not allowed to take hypoglycemic drugs until they were asked to have a meal after the colonoscopy. The bowel preparation regimens were 3-L split-dose sulfate-free polyethylene glycol (SF-PEG, Beijing Pharmaceutical Co,164.4g/bag). Patients received three bags of SF-PEG and mixed it with 3L normal-temperature mineral water in graduated bottles. The drinking rate is 250 mL every 10–15 min. Patients were instructed to drink 1 L of SF-PEG solutions at 22:00 before the day of the colonoscopy, and the remaining 2 L of SF-PEG solutions were drunk at 4–6 h before the colonoscopy.

### Quality and tolerability assessment

Colonoscopy was conducted by experienced gastrointestinal endoscopists blinded to the study grouping. The experienced endoscopists assessed the bowel cleaning quality according to Boston Bowel Preparation Scale (BBPS) (Calderwood and Jacobson, 2010). Cleanliness was evaluated for the right colon (cecum, ascending), transverse (hepatic and splenic flexures), and left colon (descending colon, sigmoid, and rectum) separately. Each colon score from 0 to 3 is defined as follows: 0) Unprepared colon segment with mucosa not seen because of solid stool that cannot be cleared; (1)Portion of the mucosa of the colon segment seen, but other areas of the colon segment not well seen because of staining, residual stool, or opaque liquid; 2) Minor amount of residual staining, small fragments of stool or opaque liquid, but mucosa of colon segment seen well; 3) Entire mucosa of colon segment seen well with no residual staining, small fragments of stool, or opaque liquid. The total score was calculated by adding the cleanliness scores of 3 segments. The total BBPS scores <6 or any segment of colon scores <2 was considered inadequate bowel cleaning. Total BBPS scores ranging from 6 to 7 were considered good bowel cleaning. Total BBPS scores ranging from 8 to 9 were considered excellent bowel cleaning.

A validated colonoscopy preparation tolerability scale (Lawrance Bowel-Preparation Tolerability Questionnaire) was used to evaluate the tolerability. All participants need complete the Lawrance questionnaire, including 9 symptoms (unpleasant taste, excessive thirst, nausea, vomiting, bloating, abdominal pain, headache, dizziness, and sleep disturbance) during bowel preparation in 5 grades. Grades for individual items are none 0), very mild 1), mild 2), moderate 3), and severe 4). Total scores are the sum of the individual item scores (Lawrance et al., 2013). In addition,7-point self-measured blood glucose (SMBG) was also conducted to assess the occurrence of hypoglycemia from administering PEG solutions to the end of the endoscopy procedure. Hypoglycemia was defined as patients’ measured blood glucose meter recording less than 3.9 mmol/L with or without low sugar symptoms and classified into 3 grades. The first-grade blood glucose ranges from 3.0 to 3.9 mmol/L. Second grade is blood glucose less than 3.0 mmol/L. Third grade is severe hypoglycemia with changes in mental status or the need for assistance to restore blood glucose.

### Statistical analysis

Data were analyzed using the SPSS 23.0 software (SPSS Inc. IBM). Descriptive data are expressed as means ± standard deviation (SD) or median (interquartile range). Discrete variables were summarized in frequency tables (N, %). Statistical differences among groups were performed with a one-way analysis of variance (ANOVA) followed by the Turkey test for multiple comparisons. The Wilcoxon rank-sum test was used for ordinal data. The chi-squared (χ2) test or Fisher exact test was used to compare categorical variables. A two-tailed value of*P*< 0.05 was considered statistically significant.

## Results

### Baseline demographic and clinical characteristics

The study population consisted of the liraglutide group (n = 120), sitagliptin group (n = 120), and control group (n = 120). The baseline demographic and clinical characteristics of participants were summarized in Table 1. Overall, 221 (61.4%) of them were male. The mean age and diabetic duration were 56.8 ± 8.1 years old and 7.4 ± 5.2 years, respectively. The baseline characteristics were similar among the three groups. There were no significant differences in age, gender, BMI, diabetic duration, HbA1c, hypertension, diabetes complications, number of other OADs or insulin, and bad habits like smoking and drinking among the three groups (all *p* > 0.05).

**TABLE 1:** Baseline demographic and clinical characteristics.

Variables	Total (n = 360)	Control group (n = 120)	Sitagliptin group (n = 120)	Liraglutide group (n = 120)	*P*
Age (year)	56.8 ± 8.1	57.1 ± 7.6	56.7 ± 7.7	56.6 ± 9.1	0.893
Male, n (%)	221 (61.4)	72 (60.0)	74 (61.7)	75 (62.5)	0.921
BMI (kg/m^2^)	24.1 ± 3.2	24.2 ± 3.3	24.4 ± 3.0	23.8 ± 3.2	0.371
Duration (year)	7.4 ± 5.2	6.7 ± 4.8	7.6 ± 6.2	7.8 ± 4.6	0.263
HbA1c (%)	8.2 ± 2.3	8.1 ± 2.2	8.3 ± 2.4	8.3 ± 2.4	0.626
Creatinine (umol/L)	67.8 ± 15.4	68.4 ± 14.7	67.6 ± 15.3	67.8 ± 16.2	0.683
ALT (U/L)	35.6 ± 11.2	35.4 ± 9.8	36.1 ± 10.3	35.7 ± 11.2	0.749
Hypertension, n (%)	146 (40.6)	49 (40.8)	47 (39.2)	50 (41.7)	0.923
Smoking, n (%)	96 (26.7)	28 (23.3)	33 (27.5)	35 (29.2)	0.575
Drinking, n (%)	88 (24.4)	27 (22.5)	29 (24.2)	32 (26.7)	0.751
DR, n (%)	61 (16.9)	19 (15.8)	22 (18.3)	20 (16.7)	0.871
DN, n (%)	42 (11.7)	13 (10.8)	15 (12.5)	14 (11.7)	0.922
DPN, n (%)	85 (23.6)	26 (21.7)	28 (23.3)	31 (25.8)	0.746
ASCVD, n (%)	31 (8.6)	9 (7.5)	12 (10.0)	10 (8.3)	0.847
Insulin, n (%)	33 (9.2)	15 (12.5)	12 (10.0)	6 (5.0)	0.122
Number of other OADs					
0	26 (7.2)	7 (5.8)	9 (7.5)	10 (8.3)	0.748
1	159 (44.2)	49 (40.8)	52 (43.3)	58 (48.3)	0.492
2	126 (35.0)	47 (39.2)	40 (33.3)	39 (32.5)	0.499
3	32 (8.9)	12 (10.0)	10 (8.3)	10 (8.3)	0.872

BMI, body mass index; HbA1c, glycated hemoglobin; ALT, alanine aminotransferase; DN, diabetic nephropathy; DR, diabetic retinopathy; DPN, diabetic peripheral neuropathy; ASCD, atherosclerosis cardiovascular disease.

### Effects of liraglutide or sitagliptin on bowel preparation quality

**FIGURE 2:**
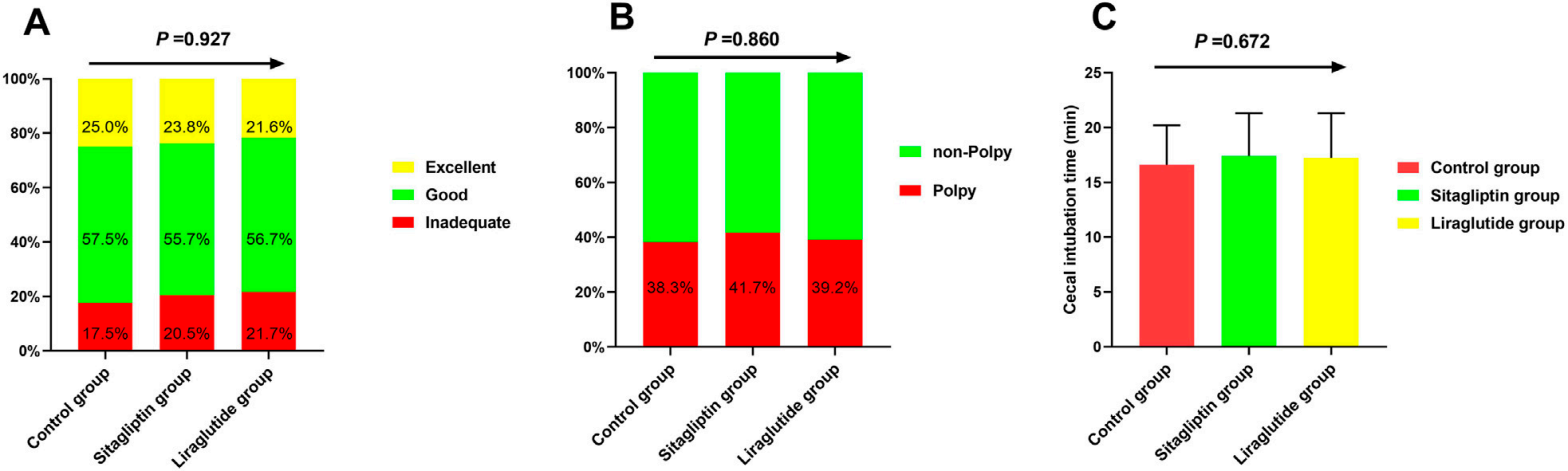
The incidence of inadequate bowel cleaning **(A)**, polyp-detecting rates **(B)**, and cecal intubation time **(C)** among the three groups.

The BBPS of the participants in the control, sitagliptin, and liraglutide groups were summarized in Table 2. The mean BBPS was 7(6–7), 2(2–2), 2(2–2), and 2 (2–3) in total, left colon, transverse colon, and right colon, respectively. There were no significant differences in any segments among the three groups (all *p* > 0.05). Figures 2A–C shows the three groups’ bowel cleaning quality, polyp detecting rate, and cecal intubation time. The incidence of adequate bowel cleaning (good and excellent) were 82.5%, 79.5%, and 78.3% in the control, sitagliptin, and liraglutide groups, respectively (Figure 2A), and the difference among the three groups was not statistically significant (*p* = 0.927). In addition, liraglutide or sitagliptin did not significantly influence the polyp-detecting rates (*p* = 0.860). The polyp-detecting rates were 38.3%,41.7%, and 39.2% (Figure 2B). Meanwhile, as shown in Figure 2C, there was no significant difference in cecal intubation time among the three groups (*p* = 0.672).

**TABLE 2:** The BBPS of the participants in the control, sitagliptin, and liraglutide groups.

Variables	Total (n = 360)	Control group (n = 120)	Sitagliptin group (n = 120)	Liraglutide group (n = 120)	*P*
Total	7 (6–7)	7 (6–7)	7 (6–7)	6 (6–7)	0.678
Left colon	2 (2–2)	2 (2–3)	2 (2–2)	2 (2–2)	0.597
Transverse colon	2 (2–2)	2 (2–2)	2 (2–2)	2 (2–2)	0.632
Right colon	2 (2–3)	2 (2–2)	2 (2–3)	2 (2–3)	0.721

BBPS, boston bowel preparation scale.

### Subgroup analyses for adequate bowel preparation

**FIGURE 3:**
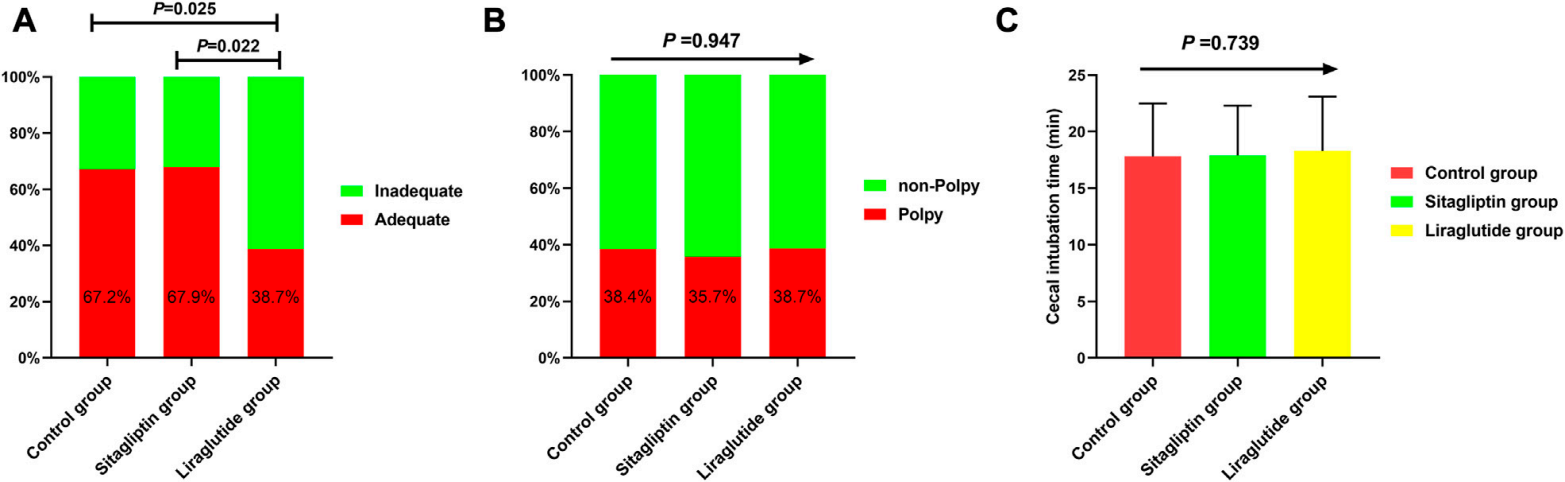
The incidence of inadequate bowel cleaning **(A)**, polyp-detecting rates **(B)**, and cecal intubation time **(C)** in T2DM with DPN among the three groups.

**FIGURE 4:**
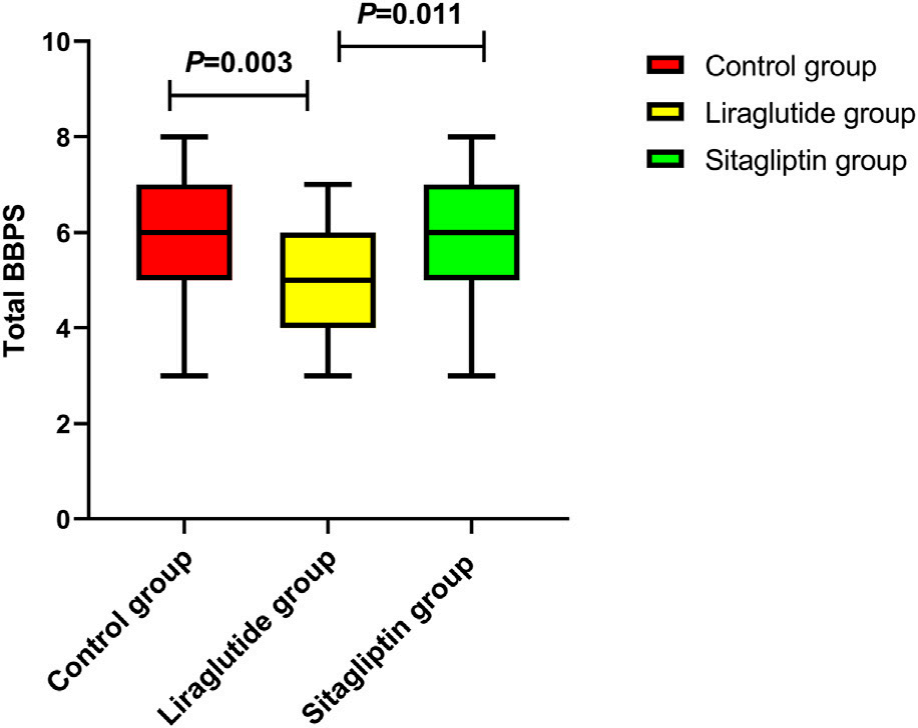
The distribution of BBPS in T2DM with DPN among the three groups.

We also performed subgroup analyses to evaluate the impacts of liraglutide or sitagliptin on bowel preparation quality. As shown in Table 3, liraglutide and sitagliptin did not significantly influence the adequate bowel preparation rate in subgroups of sex, age, diabetes duration, hypertension, DR, DN, and ASCVD (all *P* > 0.05). However, in participants with DPN, the incidence of adequate bowel cleaning in the liraglutide group was significantly decreased than in the other two groups (Figure 3A). On the contrary, there were no significant differences in the polyp-detecting rates (Figure 3B) and cecal intubation time (Figure 3C). Furthermore, the mean BBPS in the liraglutide group also significantly decreased than in the other two groups (Figure 4).

**TABLE 3:** Subgroup analyses for adequate bowel preparation.

Variables	Control group (n = 99) (n = 120)	Sitagliptin group (n = 95)	Liraglutide group (n = 94)	*P*
Sex,n (%)				
Male	59 (81.9)	58 (78.4)	58 (77.3)	0.772
Female	40 (83.3)	37 (80.4)	36 (80.0)	0.903
Age,n (%)				
<60	63 (87.5)	62 (83.8)	60 (80.0)	0.469
≥60	36 (75.0)	33 (71.7)	34 (75.6)	0.903
Diabetes duration,n (%)				
<10	64 (88.9)	62 (83.8)	63 (84.0)	0.613
≥10	35 (72.9)	33 (71.7)	31 (68.9)	0.908
Hypertension,n (%)				
With	35 (71.4)	33 (70.2)	36 (72.0)	0.981
Without	64 (90.1)	62 (84.9)	58 (82.9)	0.438
DR,n (%)				
With	12 (63.2)	15 (68.2)	13 (65.0)	0.943
Without	87 (86.1)	80 (81.6)	81 (81.0)	0.573
DN,n (%)				
With	8 (61.5)	9 (60.0)	8 (57.1)	0.972
Without	91 (85.0)	86 (81.9)	86 (81.1)	0.726
DPN,n (%)				
With	18 (67.2)	19 (67.9)	12 (38.7)	0.028
Without	81 (86.2)	76 (82.6)	82 (93.2)	0.097
ASCVD, n (%)				
With	6 (66.7)	7 (58.3)	7 (70.0)	0.839
Without	93 (83.8)	88 (81.5)	87 (79.1)	0.668

DN, Diabetic nephropathy; DR, Diabetic retinopathy; DPN, Diabetic peripheral neuropathy; ASCD, atherosclerosis cardiovascular disease.

### Effects of liraglutide or sitagliptin on bowel preparation tolerability and safety

Lawrance tolerability scores were conducted to assess gastrointestinal symptoms during bowel preparation. All participants completed the bowel preparation and colonoscopy. Table 4 shows that no serious adverse events happened among the three groups. Most adverse events were adjudicated as scores 1 or 2. In addition, no significant differences were observed in unpleasant taste, excessive thirst, abdominal pain, headache, dizziness, and sleep disturbance scores among the three groups (all *p* > 0.05). On the contrary, nausea, vomiting, and bloating scores increased in the liraglutide group compared with the other two groups (*p* < 0.05). Furthermore, few hypoglycemic events occurred in the three groups, and the differences among the three groups were not statistically significant (*p* > 0.05).

**TABLE 4:** Lawrance Tolerability Scores and hypoglycemic.

Variables	Control group (n = 120)	Sitagliptin group (n = 120)	Liraglutide group (n = 120)	*P*
Unpleasant taste	0 (0–1)	0 (0–1)	1 (0–1)	0.587
Excessive thirst	0 (0–0)	0 (0–1)	0 (0–0)	0.769
Nausea	1 (0–1)	1 (0–1)	1 (1–2)^ab^	0.008
Vomiting	0 (0–1)	0 (0–1)	1 (1–1)^ab^	0.032
Bloating	1 (0–1)	1 (0–1)	1 (1–2)^ab^	0.019
Abdominal pain	0 (0–1)	0 (0–1)	0 (0–1)	0.692
Headache	0 (0–1)	0 (0–1)	0 (0–1)	0.799
Dizziness	0 (0–0)	0 (0–0)	0 (0–0)	0.782
Sleep disturbance	1 (0–1)	0 (0–1)	1 (0–1)	0.435
Hypoglycemia				
Total, n (%)	9 (7.5)	8 (6.7)	8 (6.7)	0.958
Level 1, n (%)	6 (5.0)	5 (4.2)	6 (5.0)	0.940
Level 2, n (%)	3 (2.5)	3 (2.5)	2 (1.7)	0.880
Level 3, n (%)	0	0	0	N/A

Lawrance Tolerability Scores: 0 = none, 1 = very mild, 2 = mild, 3 = moderate and 4 = severe. Level 1 hypoglycemia: blood glucose rang from 3.0 to 3.9 mmol/L. Level 2 hypoglycemia: blood glucose less than 3.0 mmol/L. Level 3 hypoglycemia: severe hypoglycemia with changes in mental status or the need for assistance to restore blood glucose. ^a^ *P < 0.05:* Control group vs. Liraglutide group. ^b^ *P<0.05:* Sitagliptin vs. Liraglutide group.

## Discussion

Colonoscopy has been widely used as a valuable tool for screening and treating colorectal disease in T2DM, often accompanied by gastrointestinal discomfort and an increased risk of malignant colorectal tumors. Successful colonoscopy depends on good bowel preparation. Emerging evidence highlighted that GLP-1 RAs and DPP-4i profoundly affect the gastrointestinal motor system, which may influence the bowel cleaning quality. Hence, this observation study mainly assessed the effects of GLP-1RA liraglutide and DPP-4i sitagliptin on bowel cleaning quality, tolerability, and safety. The results revealed that the incidence of adequate bowel cleaning was similar among the three groups. Subgroup analyses showed that the incidence of adequate bowel cleaning in the liraglutide group was significantly decreased compared with the other two groups. Furthermore, nausea, vomiting, and bloating scores were increased in the liraglutide group than in the other two groups.

Polyethylene glycol (PEG) is widely used for bowel cleaning and is recommended as a first-line drug in Chinese bowel preparation guidelines for colonoscopy (Cancer Endoscopy Committee of China Anti-Cancer Association, 2019). Although bowel preparation strategies are the subject of many studies, the optimal PEG dose for diabetes remains uncertain. Increasing studies reported that a higher volume of solution could achieve better bowel cleansing efficacy. Meanwhile, the increased volume of solution can also influence bowel cleaning tolerability (Enestvedt et al., 2012). T2DM is often accompanied by several diabetic-related complications and poor basic status. In addition, 4-L Split-dose PEG is usually not tolerated by diabetes in clinical practice. Thus, we adopted a 3-L Split-dose PEG for bowel preparation, which was reported to be superior to 2-L PEG in bowel cleansing quality among the Chinese population in a multi-center, randomized, controlled trial (Zhang et al., 2015). T2DM was well-recognized as a risk factor for inadequate bowel cleaning and a potential reason for a repeat colonoscopy. The latest epidemiological data from a meta-analysis showed that the odds ratio (95CI) of T2DM for inadequate bowel cleaning is 1.79 (1.54–2.09). In our study, the percentage of T2DM achieved inadequate bowel cleaning is 20.0%, which was approximately two times than the previous study that used the same 3-L Split-dose PEG for bowel preparation in the healthy population (Kim et al., 2017). Among the potential mechanism of inadequate bowel preparation in diabetes, decreased intestinal transit and slowed gastric emptying may play crucial roles (Horváth et al., 2015).

Liraglutide is an analog of the human native GLP-1 hormone released from the proximal small intestinal L cells with an increased plasma half-life compared with the natural hormone. The ADA and CDS guidelines have recommended it as the first-line hypoglycemic agent for T2DM with a high risk of ASCD, chronic kidney disease, and obesity. It has been well established that GLP-1 RAs have an additional glycemic control effect beyond the pancreas by influencing gastrointestinal motility (Smits et al., 2016; Dong et al., 2021). Nakatani. Y et al. demonstrated by capsule endoscopy that GLP-1 RAs liraglutide could delay gastric emptying in T2DM and inhibit duodenal and small intestinal motility (Nakatani et al., 2017). Furthermore, another GLP-1 RAs exenatide was also reported to have a slowing effect on gastric emptying (DeFronzo et al., 2008) and small intestine motility (Thazhath et al., 2016). In contrast, the impact of GLP-1 on colonic motility was opposite compared with other intestinal segments. A recent randomized and placebo-controlled trial confirmed that liraglutide could accelerate large bowel transit and decreases the motility index in type 1 diabetes and polyneuropathy, which may indicate better coordination of propulsive motility (Wegeberg et al., 2020). Endogenous GLP-1 has a modest effect on gastric emptying. Sitagliptin is a hypoglycemic agent that can increase endogenous GLP-1 concentrations. Since they only slightly increased endogenous GLP-1 concentrations, evidence from clinical trials confirmed that DPP-4i sitagliptin, vildagliptin, and anagliptin did not influence gastric emptying (Vella et al., 2007; Rhee et al., 2014; Nakagawa et al., 2019). It appeared that the effect of GLP-1 RAs on gastric emptying decreased over time and diminished markedly over the course of 3–4 weeks (Horowitz et al., 2012; Jelsing et al., 2012). Therefore, we mainly enrolled T2DM who received liraglutide or sitagliptin within 4 weeks. Gastrointestinal motility plays an important role in bowel preparation. Decreased gastrointestinal motility is often accompanied by poor bowel cleaning quality and an increased incidence of uncomfortable abdominal symptoms. Our study showed that the percentage of participants who achieved adequate bowel cleaning was similar among the three groups. These findings are consistent with a retrospective study, which found that GLP-1 RAs likely do not contribute to the higher incidence of inadequate bowel preparation in diabetes (Sharma et al., 2017). At the same time, subgroup analyses showed that the incidence of inadequate bowel cleaning in the liraglutide group was significantly higher than in the other two groups in the DPN population. Gastrointestinal autonomic nerve damage was consistent with peripheral nerve damage in diabetes. As the degree of peripheral neuropathy deepens, the gastrointestinal electrical activity becomes more abnormal, resulting in slower gastrointestinal transmission (Farmer et al., 2017). Thus, we can speculate that the effect of liraglutide on gastrointestinal motility may further delay gastrointestinal transmission in T2DM with DPN, leading to inadequate bowel cleaning quality. These findings suggested that more attention and aggressive bowel preparation regimens should be given to this population. Based on the principle of individualized bowel preparation, a 3-L Split-dose PEG regimen combined with prokinetic or laxative agents like magnesium sulfate solution may increase the bowel preparation quality among these populations. Vomiting, nausea, and bloating were common gastrointestinal adverse reactions during liraglutide treatment (Bettge et al., 2017). The present study also revealed that the Lawrance Tolerability Scores like vomiting, nausea, and bloating were increased in liraglutide than in the other 2 groups. In comparison, most of them are mild and tolerable. Hypoglycemia is another potential adverse event to be concerned about during bowel preparation and colonoscopy. This study found that liraglutide or sitagliptin did not increase the incidence of hypoglycemia. Thus, liraglutide or sitagliptin did not influence the safety during bowel preparation and colonoscopy.

## Limitation

This study evaluated the potential effects of GLP-1 RAs liraglutide and DPP-4i sitagliptin on bowel cleaning quality, tolerability, and safety. Some limitations should be mentioned in this. First, the influence on gastrointestinal motility among GLP-1 receptor agonists may differ, and the results may not apply to other GLP-1 RAs or DPP-4i. Second, this study was designed as an observational study. Although the baseline characteristics were similar among the three groups, more randomized and placebo-controlled trials were needed to confirm these findings further.

## Conclusion

In conclusion, GLP-1RA liraglutide and DPP-4i sitagliptin did not significantly increase the incidence of inadequate bowel cleaning. Meanwhile, liraglutide and sitagliptin do not affect the tolerability and safety during bowel preparation and colonoscopy. By contrast, liraglutide increase the incidence of inadequate bowel preparation in T2DM with DPN. This study reveal that more attention and aggressive bowel preparation regimens should be given to the T2DM with DPN.

## Data Availability

The original contributions presented in the study are included in the article/Supplementary Material, further inquiries can be directed to the corresponding authors.

## References

[B1] Aleksandrova K. Boeing H. Jenab M. Bas Bueno-de-Mesquita H. Jansen E. van Duijnhoven F. J. B. (2011). Metabolic syndrome and risks of colon and rectal cancer: The European prospective investigation into cancer and nutrition study. Cancer Prev. Res. 4 (11), 1873–1883. 10.1158/1940-6207.CAPR-11-0218 21697276

[B2] Bettge K. Kahle M. Abd El Aziz M. Meier J. J. Nauck M. A. (2017). Occurrence of nausea, vomiting and diarrhoea reported as adverse events in clinical trials studying glucagon-like peptide-1 receptor agonists: A systematic analysis of published clinical trials. Diabetes, Obes. metab. 19 (3), 336–347. 10.1111/dom.12824 27860132

[B3] Calderwood A. Jacobson B. (2010). Comprehensive validation of the Boston bowel preparation scale. Gastrointest. Endosc. 72 (4), 686–692. 10.1016/j.gie.2010.06.068 20883845 PMC2951305

[B4] Cancer Endoscopy Committee of China Anti-Cancer Association (2019). Chinese guideline for bowel preparation for colonoscopy (2019, Shanghai) [J]. Zhonghua nei ke za zhi 58 (7), 485–495. 10.3760/cma.j.issn.0578-1426.2019.07.002 31269564

[B5] Chung Y. Han D. Park K. Kim K. O. Park C. H. Hahn T. (2009). Patient factors predictive of inadequate bowel preparation using polyethylene glycol: A prospective study in korea. J. Clin. gastroenterology 43 (5), 448–452. 10.1097/MCG.0b013e3181662442 18978506

[B6] Deacon C. (2020). Dipeptidyl peptidase 4 inhibitors in the treatment of type 2 diabetes mellitus. Nat. Rev. Endocrinol. 16 (11), 642–653. 10.1038/s41574-020-0399-8 32929230

[B7] DeFronzo R. Okerson T. Viswanathan P. Guan X. Holcombe J. H. MacConell L. (2008). Effects of exenatide versus sitagliptin on postprandial glucose, insulin and glucagon secretion, gastric emptying, and caloric intake: A randomized, cross-over study. Curr. Med. Res. Opin. 24 (10), 2943–2952. 10.1185/03007990802418851 18786299

[B8] Dong Y. Carty J. Goldstein N. He Z. Hwang E. Chau D. (2021). Time and metabolic state-dependent effects of GLP-1R agonists on NPY/AgRP and POMC neuronal activity *in vivo* . Mol. Metab. 54, 101352. 10.1016/j.molmet.2021.101352 34626854 PMC8590079

[B9] Drucker D. (2018). Mechanisms of action and therapeutic application of glucagon-like peptide-1. Cell metab. 27 (4), 740–756. 10.1016/j.cmet.2018.03.001 29617641

[B10] Enestvedt B. Tofani C. Laine L. Tierney A. Fennerty M. B. (2012). 4-Liter split-dose polyethylene glycol is superior to other bowel preparations, based on systematic review and meta-analysis. Clin. Gastroenterol. Hepatol. 10 (11), 1225–1231. 10.1016/j.cgh.2012.08.029 22940741

[B11] Farmer A. D. Pedersen A. G. Brock B. Jakobsen P. E. Karmisholt J. Mohammed S. D. (2017). Type 1 diabetic patients with peripheral neuropathy have pan-enteric prolongation of gastrointestinal transit times and an altered caecal pH profile. Diabetologia 60 (4), 709–718. 10.1007/s00125-016-4199-6 28105520

[B12] Gandhi K. Tofani C. Sokach C. Patel D. Kastenberg D. Daskalakis C. (2018). Patient characteristics associated with quality of colonoscopy preparation: A systematic review and meta-analysis. Clin. Gastroenterol. Hepatol. 16 (3), 357–369.e10. 10.1016/j.cgh.2017.08.016 28826680

[B13] Horowitz M. Flint A. Jones K. Hindsberger C. Rasmussen M. F. Kapitza C. (2012). Effect of the once-daily human GLP-1 analogue liraglutide on appetite, energy intake, energy expenditure and gastric emptying in type 2 diabetes. Diabetes Res. Clin. Pract. 97 (2), 258–266. 10.1016/j.diabres.2012.02.016 22446097

[B14] Horváth V. Putz Z. Izbéki F. Korei A. E. Gerő L. Lengyel C. (2015). Diabetes-related dysfunction of the small intestine and the colon: Focus on motility. Curr. diabetes Rep. 15 (11), 94. 10.1007/s11892-015-0672-8 26374571

[B15] Jalleh R. Jones K. Rayner C. Marathe C. S. Wu T. Horowitz M. (2022). Normal and disordered gastric emptying in diabetes: Recent insights into (patho)physiology, management and impact on glycaemic control. Diabetologia 65 (12), 1981–1993. 10.1007/s00125-022-05796-1 36194250 PMC9630190

[B16] Jelsing J. Vrang N. Hansen G. Raun K. Tang-Christensen M. Knudsen L. B. (2012). Liraglutide: Short-lived effect on gastric emptying - long lasting effects on body weight. Diabetes, Obes. metab. 14 (6), 531–538. 10.1111/j.1463-1326.2012.01557.x 22226053

[B17] Kim Y. Seo E. Lee J. Lee S. H. Park H. S. Choi S. H. (2017). Inadequate bowel cleansing efficacy of split-dose polyethylene glycol for colonoscopy in type 2 diabetic patients: A prospective and blinded study. J. Clin. gastroenterology 51 (3), 240–246. 10.1097/MCG.0000000000000536 27136960

[B18] Lawrance I. Willert R. Murray K. (2013). A validated bowel-preparation tolerability questionnaire and assessment of three commonly used bowel-cleansing agents. Dig. Dis. 58 (4), 926–935. 10.1007/s10620-012-2449-0 23095990

[B19] Marathe C. Rayner C. Jones K. Michael H. (2011). Effects of GLP-1 and incretin-based therapies on gastrointestinal motor function. Exp. diabetes Res. 2011, 279530. 10.1155/2011/279530 21747825 PMC3124003

[B20] Nakagawa T. Nagai Y. Yamamoto Y. Miyachi A. Hamajima H. Mieno E. (2019). Effects of anagliptin on plasma glucagon levels and gastric emptying in patients with type 2 diabetes: An exploratory randomized controlled trial versus metformin. Diabetes Res. Clin. Pract. 158, 107892. 10.1016/j.diabres.2019.107892 31669625

[B21] Nakatani Y. Maeda M. Matsumura M. Shimizu R. Banba N. Aso Y. (2017). Effect of GLP-1 receptor agonist on gastrointestinal tract motility and residue rates as evaluated by capsule endoscopy. Diabetes & metabolism 43 (5), 430–437. 10.1016/j.diabet.2017.05.009 28648835

[B22] Nauck M. Niedereichholz U. Ettler R. Holst J. J. Orskov C. Ritzel R. (1997). Glucagon-like peptide 1 inhibition of gastric emptying outweighs its insulinotropic effects in healthy humans. Am. J. physiology 273 (5), E981–E988. 10.1152/ajpendo.1997.273.5.E981 9374685

[B23] Ogurtsova K. Guariguata L. Barengo N. Ruiz P. L. D. Sacre J. W. Karuranga S. (2022). IDF diabetes Atlas: Global estimates of undiagnosed diabetes in adults for 2021. Diabetes Res. Clin. Pract. 183, 109118. 10.1016/j.diabres.2021.109118 34883189

[B24] Rhee N. Østoft S. Holst J. Deacon C. F. Vilsboll T. Knop F. K. (2014). The impact of dipeptidyl peptidase 4 inhibition on incretin effect, glucose tolerance, and gastrointestinal-mediated glucose disposal in healthy subjects. Eur. J. Endocrinol. 171 (3), 353–362. 10.1530/EJE-14-0314 24935932

[B25] Sang M. Wu T. Zhou X. Horowitz M. Jones K. L. Qiu S. (2022). Prevalence of gastrointestinal symptoms in Chinese community-dwelling adults with and without diabetes. Nutrients 14 (17), 3506. 10.3390/nu14173506 36079764 PMC9459935

[B26] Selbuz S. Buluş A. (2020). Gastrointestinal symptoms in pediatric patients with type 1 diabetes mellitus. J. Pediatr. Endocrinol. metabolism 33 (2), 185–190. 10.1515/jpem-2019-0350 31846427

[B27] Sharma T. Das N. Ismail B. Castro-Pavia F. Cabral J. Villabona C. (2017). Evaluation of the effect of GLP-1 agonists on quality of bowel preparation for colonoscopy in patients with diabetes. Pract. diabetes 34 (5), 167–168. 10.1002/pdi.2110

[B28] Smits M. Tonneijck L. Muskiet M. Kramer M. H. H. Cahen D. L. van Raalte D. H. (2016). Gastrointestinal actions of glucagon-like peptide-1-based therapies: Glycaemic control beyond the pancreas. Diabetes, Obes. metab. 18 (3), 224–235. 10.1111/dom.12593 26500045

[B29] Thazhath S. Marathe C. Wu T. Chang J. Khoo J. Kuo P. (2016). The glucagon-like peptide 1 receptor agonist exenatide inhibits small intestinal motility, flow, transit, and absorption of glucose in healthy subjects and patients with type 2 diabetes: A randomized controlled trial. Diabetes 65 (1), 269–275. 10.2337/db15-0893 26470783

[B30] Vella A. Bock G. Giesler P. Burton D. B. Serra D. B. Saylan M. L. (2007). Effects of dipeptidyl peptidase-4 inhibition on gastrointestinal function, meal appearance, and glucose metabolism in type 2 diabetes. Diabetes 56 (5), 1475–1480. 10.2337/db07-0136 17303799

[B31] Wang J. Wang Q. Yang X. Yang W. Li D. R. Jin J. Y. (2023). GLP-1 receptor agonists for the treatment of obesity: Role as a promising approach. Front. Endocrinol. 14, 1085799. 10.3389/fendo.2023.1085799 PMC9945324 36843578

[B32] Wegeberg A. Hansen C. Farmer A. Karmisholt J. S. Drewes A. M. Jakobsen P. E. (2020). Liraglutide accelerates colonic transit in people with type 1 diabetes and polyneuropathy: A randomised, double-blind, placebo-controlled trial. United Eur. gastroenterology J. 8 (6), 695–704. 10.1177/2050640620925968 PMC7437086 32390563

[B33] Zhang S. Li M. Zhao Y. Lv T. Shu Q. Zhi F. (2015). 3-L split-dose is superior to 2-L polyethylene glycol in bowel cleansing in Chinese population: A multicenter randomized, controlled trial. Medicine 94 (4), e472. 10.1097/MD.0000000000000472 25634195 PMC4602972

